# Trophic ecology of Angolan cold-water coral reefs (SE Atlantic) based on stable isotope analyses

**DOI:** 10.1038/s41598-023-37035-x

**Published:** 2023-06-19

**Authors:** Beatriz Vinha, Sergio Rossi, Andrea Gori, Ulrike Hanz, Antonio Pennetta, Giuseppe E. De Benedetto, Furu Mienis, Veerle A. I. Huvenne, Dierk Hebbeln, Claudia Wienberg, Jürgen Titschack, André Freiwald, Stefano Piraino, Covadonga Orejas

**Affiliations:** 1 Dipartimento di Scienze e Tecnologie Biologiche e Ambientali (DiSTeBA), Università del Salento, 73100 Lecce, Italy; 2 Hanse Wissenschaftskolleg – Institute for Advanced Study, 27753 Delmenhorst, Germany; 3 Instituto de Ciências Do Mar, LABOMAR, Universidade Federal do Ceará, Fortaleza, 60165-081 Brazil; 4 CoNISMa, Consorzio Nazionale Interuniversitario per le Scienze del Mare, 00196 Rome, Italy; 5 Departament de Biologia Evolutiva, Ecologia i Ciències Ambientals, Universitat de Barcelona, 08028 Barcelona, Spain; 6 Institut de Recerca de La Biodiversitat (IRBio), Universitat de Barcelona, 08028 Barcelona, Spain; 7 Department of Ocean Systems, NIOZ Royal Netherlands Institute for Sea Research, Texel, 1790AB the Netherlands; 8 Bentho-Pelagic Processes, Alfred Wegener Institute for Polar and Marine Research, 27570 Bremerhaven, Germany; 9 Laboratorio di Spettrometria di Massa Analitica e Isotopica, Dipartimento di Beni Culturali, Università del Salento, 73100 Lecce, Italy; 10 Ocean BioGeosciences, National Oceanography Centre, Southampton, S014 3ZH UK; 11 MARUM – Center for Marine Environmental Sciences, University of Bremen, 28359 Bremen, Germany; 12 Senckenberg Am Meer, Marine Research Department, 26382 Wilhelmshaven, Germany; 13 NBFC, National Biodiversity Future Center, 90133 Palermo, Italy; 14 Instituto Español de Oceanografía, Centro Oceanográfico de Gijón, (IEO-CSIC), 33212 Gijón, Spain

**Keywords:** Marine biology, Stable isotope analysis, Food webs

## Abstract

Cold-water coral (CWC) reefs of the Angolan margin (SE Atlantic) are dominated by *Desmophyllum pertusum* and support a diverse community of associated fauna, despite hypoxic conditions. In this study, we use carbon and nitrogen stable isotope analyses (δ^13^C and δ^15^N) to decipher the trophic network of this relatively unknown CWC province. Although fresh phytodetritus is available to the reef, δ^15^N signatures indicate that CWCs (12.90 ± 1.00 ‰) sit two trophic levels above Suspended Particulate Organic Matter (SPOM) (4.23 ± 1.64 ‰) suggesting that CWCs are highly reliant on an intermediate food source, which may be zooplankton. Echinoderms and the polychaete *Eunice norvegica* occupy the same trophic guild, with high δ^13^C signatures (-14.00 ± 1.08 ‰) pointing to a predatory feeding behavior on CWCs and sponges, although detrital feeding on ^13^C enriched particles might also be important for this group. Sponges presented the highest δ^15^N values (20.20 ± 1.87 ‰), which could be due to the role of the sponge holobiont and bacterial food in driving intense nitrogen cycling processes in sponges’ tissue, helping to cope with the hypoxic conditions of the reef. Our study provides first insights to understand trophic interactions of CWC reefs under low-oxygen conditions.

## Introduction

Cold-water corals (CWCs) are heterotrophic cnidarians with an opportunistic suspension-feeding behavior^1^. In the food-limited deep sea, carbon processing in CWC reefs is higher compared to other adjacent habitats^2,3^ and trophic interactions are one of the processes contributing to the transfer and cycling of organic carbon between different functional groups within the reef^2^. Topography-enhanced hydrodynamics^4–7^ and vertical downwelling bringing surface waters to the deep^8^ are some of the mechanisms responsible for the enhanced transport of organic carbon  to CWC reefs, driving CWC occurrence and distribution^9–11^.

The CWC *Desmophyllum pertusum*, also known as *Lophelia pertusa*^12^, forms biogenic frameworks with three-dimensional complexity^13,14^ representing a model of ecosystem engineering in the deep sea^15^. As for other framework-forming CWCs, *D. pertusum* reefs increase the local biodiversity of associated fauna^16–20^ by (i) providing a complex habitat with multiple ecological niches in areas of enhanced hydrodynamics favoring increased food supply^21,22^ and (ii) by locally reducing flow velocity and turbulence in the shadowed central zones of the reef improving food capture^23,24^ and the settlement of larvae^23^.

Thriving CWC reefs dominated by *D. pertusum*^25^, with the presence of *Madrepora oculata*^26^*,* have recently been found between 331 and 473 m water depth along the Angolan margin (SE Atlantic), coinciding with the center of the local oxygen minimum zone (OMZ). The discovery of the Angolan *D. pertusum* reefs challenged some of the previously assumed ecological requirements for the species^27,28^, given that CWCs were found in very low oxygen concentrations (0.5 to 1.3 mL L^−1^) and relatively high temperatures (6.8 to 14.2 °C)^11,25,26^. Despite the hypoxic conditions, the Angolan CWC reefs are prosperous and harbor a community of associated mega- and macrofauna, mainly composed of sponges, octocorals, and antipatharians^29,30^. Most studies on the trophic ecology of CWCs are available for communities occurring in normoxic conditions in the Mediterranean Sea^31–33^ and in the North Atlantic Ocean^34–38^, with scarce information for other deep-sea regions. To date, no trophic studies have been conducted in the recently discovered reefs of Angola.

Previous works showed that *D. pertusum* is a passive suspension-feeder, capable of feeding on different food sources, including dissolved organic matter (DOM)^39,40^, particulate organic matter (POM) in the form of phytodetritus from surface primary production^37,41^ and zooplankton^31,34,42–45^. In the Angolan CWC reefs, high-quality and abundant organic matter (OM) resulting from a productive upwelling system^46^ is available to benthic communities^11^. This abundant food supply could be a key mechanism for CWC survival under the Angolan OMZ^11,47–49^. A recent study by Gori et al.^50^, showed that *D. pertusum* in Angola can maintain high respiration rates under deoxygenation, suggesting acclimation and local adaptation of the species to the environmental conditions of the reefs. Some of the proposed adaptations of benthic organisms to hypoxia might include changes in body size and shape^47,49^, changes in energetic metabolic pathways^49,50^ as well as symbiotic associations with anoxic-bearing bacteria^51–53^.  As far as we know, the effect of OMZs in the trophic ecology of CWCs and associated fauna has not yet been described. However, previous studies of trophic webs in OMZs showed the importance of microbe-mediated trophic processes^54,55^ in delivering OM to benthic organisms under low-oxygen conditions^56^, in particular, through the role of nitrifying and denitrifying bacteria^55,57^. Therefore, investigating the trophic interactions in the Angolan CWC reefs could provide new understanding of the food sources available to these communities, and how CWC reef trophic webs in OMZs might differ from those in more oxygenated regions.

Because of their remoteness and therefore the difficulty to obtain samples or conduct in situ experiments, disentangling energy flows within CWC ecosystems is challenging; nonetheless, different methodologies may allow to overcome the inherent difficulties. Gut content analysis is a widely used technique to decipher the diet of both terrestrial and marine organisms and has also been used for deep-sea animals^58,59^. However, data on the gastrovascular content of CWCs are very rare^60^ because of the complications in identifying preys due to the small size of the food particles^61^ and the difficulties in assessing the gut content by dissection of the corals’ polyps (in particular the ones from scleractinian corals). In situ detection of feeding interactions is possible through visual observations^62,63^, but such interactions are not easily captured on camera, and a large amount of data, over a large temporal scale, is needed. For these reasons, carbon (δ^13^C) and nitrogen (δ^15^N) stable isotope analyses have become the most widely used tools to investigate the trophic structures of deep-sea communities^64–70^. Stable isotope signals do not decay over time, thus integrating long-term diets and giving information on the dietary choices of an organism over a wide temporal range (from days to months, depending on the tissue turnover)^71^. In a standard food web, δ^15^N increases in a stepwise manner from one trophic level to the next, usually resulting in an enrichment of 3.4–5.0 ‰ in δ^15^N^72^ from the prey to consumer, thus, allowing to estimate the trophic position of the different organisms^73^. In addition, there is a small enrichment in δ^13^C of approximately 1 ‰ from one trophic level to the next^74^. Furthermore, the δ^13^C signature provides information of the primary source of the OM supporting the trophic web^75^. For example, enriched ^13^C ratios point to photosynthetic production (e.g., from − 22 to − 14 ‰^31^), while more depleted δ^13^C values suggest a trophic web fueled by chemoautotrophy (e.g., from − 50 to − 15 ‰ in hydrothermal and methane seep communities^76^).

In this study, we provide a first insight in the trophic structure of the Angolan CWC reefs, using stable isotope analyses. This work is a pivotal step to understand the trophic interactions of CWC reefs under hypoxic conditions and will contribute with new baseline knowledge on the ecological functioning of a poorly investigated deep-sea area of the SE Atlantic Ocean. This could shed light on how CWCs and associated deep-sea communities might cope with the forecasted impacts of climate change, specifically with the expected expansion of OMZs in the future^77^.

## Results

**Figure 1:**
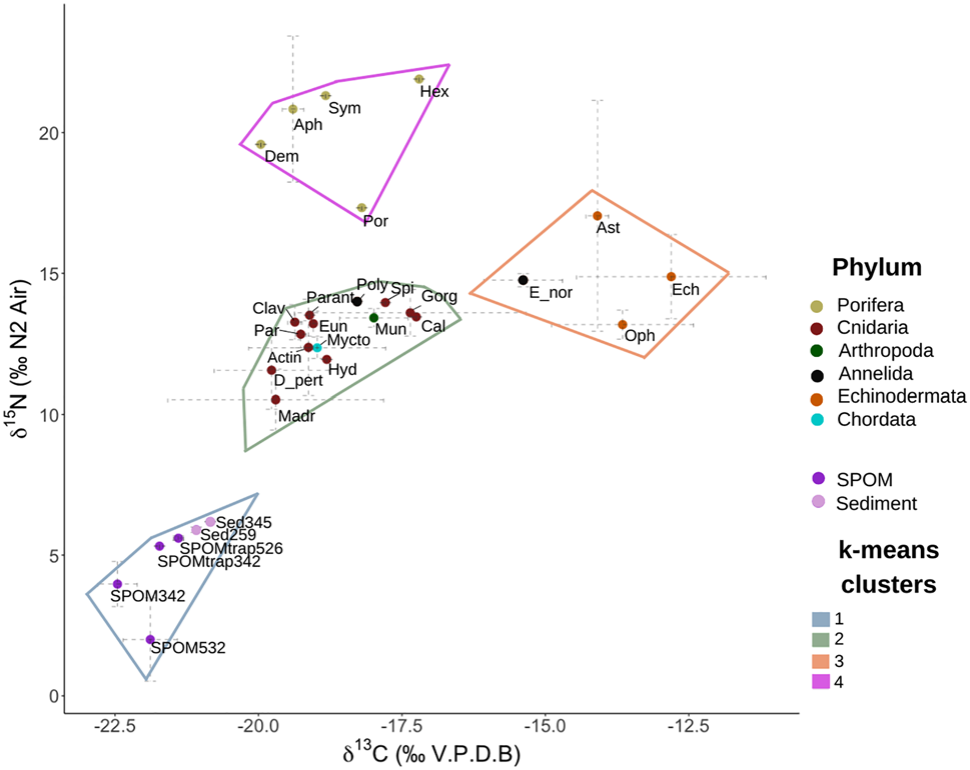
Bi-plot of the mean carbon (δ^13^C) and nitrogen (δ^15^N) stable isotopes of the main benthic megafauna groups on the Angola CWC reefs and the analyzed particulate organic matter, sampled at different depths: suspended particulate organic matter (SPOM), settling SPOM collected with a sediment trap (SPOMtrap) and sediment. Dashed grey lines represent standard deviation. Polygons represent k-means clusters, with the optimal number of clusters decided based on the elbow, silhouette and gap statistic methods. Labels in the bi-plot represent the abbreviations of the different taxonomic groups, found in Table 2. The values presented here for SPOM342, SPOM532, SPOMtrap342 and SPOMtrap526 were previously published by Hanz et al.^9^.

Carbon and nitrogen stable isotope (δ^13^C and δ^15^N) analyses were conducted on benthic megafauna samples and on three types of Particulate Organic Matter (POM; filtered Suspended Particulate Organic Matter (SPOM), SPOM from a sediment trap and POM from sediment) collected from the Angolan CWC reefs. The lowest measured δ^13^C value was for SPOM sampled at 342 m water depth (− 22.46 ± 0.34 ‰) and the highest was for *Echinus* sp. (− 12.81 ± 1.65 ‰), while δ^15^N values ranged from 2.00 ± 1.48 ‰ for SPOM sampled at 532 m water depth to 21.90 ‰ in a Hexactinellid sponge. The carbon and nitrogen stable isotope bi-plot (Fig. 1) represents a total isotopic range of 9.65 ‰ for δ^13^C and of 19.9 ‰ for δ^15^N, with a correlation of R = 0.74 (p-value < 2.2 × 10^–16^) between δ^13^C and δ^15^N. The four clusters obtained with the k-means analysis match with the trophic position (TP) calculations (Table 1), indicating that each cluster represents a different trophic guild in the Angolan CWC reefs.

**Table 1:** Mean ratios of δ^13^C and δ^15^N (± standard deviation (s.d.)) and number of samples (n) analyzed for each type of particulate organic matter and taxonomic group, with information of the label used in Fig. 1.

Taxonomic group		Label	n	δ^15^N (± s.d.) (‰)	δ^13^C (± s.d.) (‰)	TP (± s.d.)
Particulate Organic Matter (POM)	Sediment 259 m	Sed259	1	5.90	− 21.08	
	Sediment 345 m	Sed345	1	6.19	− 20.84	
	Sediment trap material 342 m	SPOMtrap342	1	5.32	− 21.73	
	Sediment trap material 526 m	SPOMtrap526	1	5.61	− 21.40	
	Suspended Particulate Organic Matter 342 m	SPOM342	20	3.97 ± 0.80	− 22.46 ± 0.34	
	Suspended Particulate Organic Matter 532 m	SPOM532	24	2.00 ± 1.48	− 21.89 ± 0.47	
Porifera	Aphrocallistes sp	Aph	3	20.83 ± 2.59	− 19.40 ± 0.19	5.88 ± 0.76
	Sympagella sp.	Sym	1	21.31	− 18.83	6.02
	Demospongiae sp.	Dem	1	19.58	− 19.96	5.51
	Hexactinellida sp.	Hex	1	21.90	− 17.20	6.20
	Porifera *indet* sp.	Por	1	17.33	− 18.20	4.85
Cnidaria	Actiniaria spp.	Actin	3	12.37 ± 1.71	− 19.13 ± 0.08	3.39 ± 0.50
	Callogorgia sp.	Cal	1	13.45	− 17.25	3.71
	Clavularia sp.	Clav	3	13.26 ± 0.62	− 19.37 ± 0.12	3.66 ± 0.18
	Eunicella sp.- −	Eun	2	13.21 ± 0.44	− 19.04 ± 0.05	3.64 ± 0.13
	Gorgoniidae spp.	Gorg	4	13.60 ± 0.83	17.35 ± 2.02	3.75 ± 0.24
	Hydrozoa	Hyd	1	11.94	− 18.81	3.27
	*Desmophyllum pertusum*	D_pert	4	11.56 ± 1.37	− 19.77 ± 1.00	3.16 ± 04.0
	*Madrepora oculata*	Madr	2	10.52 ± 1.07	− 19.70 ± 1.88	2.85 ± 0.32
	*Paramuricea* sp.	Par	3	12.83 ± 0.48	− 19.26 ± 0.09	3.52 ± 0.14
	*Parantipathes* sp.	Parant	1	13.51	− 19.11	3.73
	*Spinimuricea* sp.	Spi	1	13.96	− 17.79	3.86
Arthropoda	*Munida* sp.	Mun	2	13.41 ± 0.33	− 17.99 ± 0.59	3.70 ± 0.10
Annelida	*Eunice norvegica*	E_nor	2	14.75 ± 0.23	− 15.39 ± 0.69	4.10 ± 0.07
	Polynoidae sp.	Poly	1	14.00	− 18.28	3.87
Echinodermata	Asteroidea spp.	Ast	3	17.04 ± 4.10	− 14.09 ± 0.20	4.77 ± 1.21
	Echinus sp.	Ech	3	14.88 ± 1.49	− 12.81 ± 1.65	4.13 ± 0.44
	Ophiuroidea	Oph	2	13.18 ± 0.52	− 13.65 ± 1.24	3.63 ± 0.15
Chordata	Myctophidae sp.	Mycto	3	12.35 ± 0.38	− 18.98 ± 1.19	3.39 ± 0.11

For consumers, mean trophic position (TP) (± s.d.) was calculated following Post^72^.

The three types of POM analyzed are included in cluster 1, presenting a mean stable isotopic composition of − 21.57 ± 0.59 ‰ and 4.83 ± 1.58 ‰ for δ^13^C and δ^15^N, respectively. Within this cluster, sediment sampled at 345 m water depth presented the most enriched values (− 20.84 ‰ for δ^13^C and 6.19 ‰ for δ^15^N), being 4.19 ‰ more enriched, in terms of δ^15^N, than SPOM sampled at 532 m water depth. The two types of SPOM analyzed (filtered SPOM and SPOM from the trap), presented a mean δ^13^C value of − 21.87 ± 0.44 ‰ and a mean δ^15^N value of 4.23 ± 1.64 ‰, while sediment had more enriched mean values of − 20.96 ± 0.17 ‰ for δ^13^C and of 6.05 ± 0.21 ‰ for δ^15^N. The SPOM samples collected at deeper sites present more enriched mean δ^13^C signatures (− 21.65 ± 0.35 ‰) than the ones collected at shallower sites (− 22.10 ± 0.52).

The stable isotopic signature of consumers ranged from 10.52 ± 1.07 ‰ to 21.9 ‰ in δ^15^N, corresponding to *M. oculata* and a hexactinellid sponge, respectively, whereas the consumer with most the δ^13^C-depleted signature was *D. pertusum* (− 19.77 ± 1.0 ‰) and with the most δ^13^C-enriched signature was *Echinus* sp. (− 12.81 ± 1.65 ‰).

The consumers included in cluster 2 varied from − 19.77 ± 1.0 ‰ to − 17.25 ‰, in terms of δ^13^C and from 10.52 ± 1.07 ‰ to 14.00 ‰, in terms of δ^15^N, presenting a mean δ^13^C value of − 18.70 ± 0.83 ‰ and a mean δ^15^N value of 12.90 ± 1.00 ‰. The mean calculated TP of this cluster is 3.54 ± 0.29, with the maximum TP (3.87) occupied by a polynoid annelid (*Polynoidae* sp.). The consumers included in this cluster are all analyzed CWC species as well as the above-mentioned annelid (*Polynoidae* sp.), a hydrozoan (unidentified), a sea anemone (Actinaria) and a fish (*Myctophidae* sp.). The distance between the mean stable isotope ratios of cluster 1 (POM) to cluster 2 is 2.9 ‰ for δ^13^C and 8.07 ‰ for δ^15^N, which corresponds to 2.37 trophic levels, if a trophic enrichment for δ^15^N of 3.4 ‰ is considered.

All the echinoderm taxa analyzed, plus the polychaete *Eunice norvegica* are part of cluster 3. This group presents the most enriched carbon stable isotope signatures with mean δ^13^C of − 14.00 ± 1.08 ‰ and mean δ^15^N of 15.00 ± 1.59 ‰. The mean TP of this group is 4.16 ± 0.47.

The organism most enriched in δ^15^N was a glass sponge (*Hexactinellida* sp.; 21.90 ‰). All analyzed sponge taxa (cluster 4) presented the highest mean δ^15^N ratios (mean 20.20 ± 1.87 ‰) consequently corresponding, according to the TP calculations, to the highest mean TP in the trophic web (mean TP of 5.69 ± 0.53).

## Discussion

**Figure 2:**
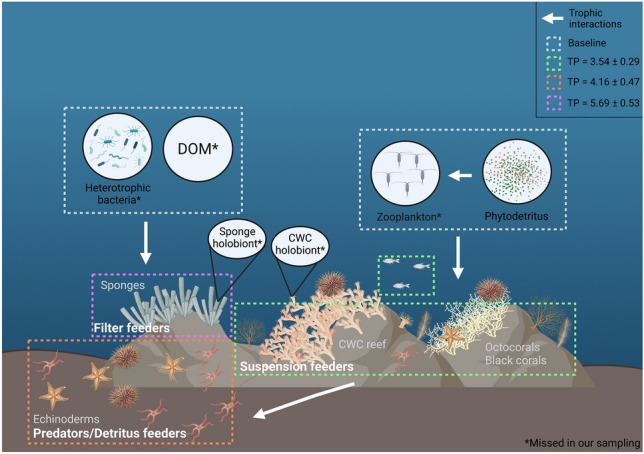
Schematic representation of the proposed trophic web for the Angolan cold-water coral (CWC) reefs. Trophic groups are based on carbon and nitrogen stable isotope (δ^13^C and δ^15^N) analyses. Mean trophic position (TP) (± s.d.) is indicated for each group as well as the proposed food sources. Created with BioRender.com.

The basic structure of the food web of CWC reefs offshore Angola was deciphered, for the first time. Our results indicate a reef trophic web composed of four different trophic guilds (Fig. 2). The carbon isotope signatures of POM (− 22.46 to − 20.84 ‰) indicate that the CWC reefs are sustained by photosynthesis-derived primary production from the photic zone, consistent with what was first shown by Le Guilloux et al*.*^78^; nonetheless, the gap of one trophic level between POM (cluster 1) and suspension feeders (cluster 2) suggests that an additional food source was missed in our sampling. Although the significant correlation between δ^13^C and δ^15^N indicates a network supported by a single primary source^65,79,80^, the enriched δ^13^C signatures of predators and detritus-feeders (− 15.39 to − 12.81 ‰) and the high δ^15^N values of sponges (17.33 to 21.90 ‰) point to different food sources for the consumers at the Angolan CWC reefs.

The Angolan basin is an area with exceptionally high primary productivity, derived from the Benguela upwelling system^46,81^, and fueled by nutrients delivered by the Cuanza and Congo Rivers^82^. It has been argued that a nutritive food source delivered by the upwelling system could play an important role in allowing the corals to tolerate the potential stress that hypoxia and high temperatures off Angola could cause^25,26,83,84^, since an abundant and easily digestible food source could help balance the energetic metabolic demands of species under oxygen stress^47,48^. Indeed, Hanz et al.^11^, showed that high-quality and abundant OM, in the form of fresh phytodetritus, is available to the CWC reefs, corroborated by the low isotopic signature of δ^15^N of SPOM, as well as the low C/N ratios and the little degraded phytopigments of the analyzed OM^11^. In addition, fluvial input of terrestrial OM slightly enriches the δ^13^C signature of the SPOM and sediment (− 22.46 to − 21.40 ‰)^11^. Our results show that *D. pertusum* and *M. oculata* are 2 ‰ enriched compared to the mean δ^13^C signature of the analyzed POM (see cluster 1, Fig. 1), indicating that possibly the corals do not directly feed on the terrigenous-enriched POM. However, the high input of nutrients associated with the terrestrial material transported with the riverine discharges could, instead, be important to maintain the primary productivity that supports the baseline of the trophic web^85^. The OM fluxes to the Angolan basin fluctuate seasonally^86^, because of seasonal changes in the upwelling system and riverine discharges^87^. It is possible that this seasonality is also reflected in the diet of the CWCs, with the corals exploiting fresh phytodetritus, when available, but also opportunistically feeding on other types of food, when fresher phytodetritus is absent^1,45,88^.

At the Angolan CWC reefs, *D. pertusum* and *M. oculata* presented slightly higher δ^15^N values (11.56 ± 1.37 ‰ and 10.52 ± 1.07 ‰, respectively) than the ones reported for other CWC reefs (7.6 to 10.1 ‰ for *D. pertusum* and 6.9 to 10 ‰ for *M. oculata*) in the NE Atlantic Ocean^34,35,37,89^ and the Mediterranean Sea^31^, even though the δ^15^N signature of SPOM in our study (2.00 to 5.32 ‰) is comparable to the ones reported in literature (2.2 to 8.3 ‰^31,34,35,37,41,44,79^). In our study area in the SE Atlantic Ocean, δ^15^N ratios show an enrichment of 7.33 ‰ and 6.29 ‰ between SPOM and *D. pertusum* and *M. oculata*, respectively. In other studies, the observed δ^15^N enrichment between *D. pertusum* and SPOM ranged from 2.7 to 7.1 ‰^31,34,35,37,44^. The observed isotopic enrichment at the Angolan reefs is only similar to the one observed in the Galicia Bank (7.1 ‰^34^) where the authors could not define a conclusive food source for the CWCs, suggesting a mixture of different sources^34^. In fact, isotopic enrichment might differ between deep-sea environments and conditions^35,44,70^ depending on the available food sources, the metabolic rate of organisms and the isotopic composition of the surrounding water column. Furthermore, the observed δ^15^N enrichment in our study could also point to the presence of an additional trophic level between the phytodetritus and the corals, missed in our sampling. This gap could indicate that, in addition to fresh phytodetritus, zooplankton might be an important food source for the CWCs offshore Angola, as previously observed in other CWC reefs dominated by scleractinians^31,34,89,90^. Indeed, zooplankton is a nutritional rich food source for cold-water scleractinians^45,91^, octocorals^92,93^, and antipatharians^31,33,94^. The Angolan basin harbors abundant and diverse zooplankton communities^95^ whose dial vertical migrations coincide with the water depth where CWCs are found (331 to 473 m)^96^. The zooplankton δ^15^N ratios (ranging from 3.5 to 10 ‰) from other regions of the Atlantic sampled in the same depth range (300 to 500 m) as our study^34,37,97–99^ fit with the expected enrichment of the intermediate trophic level. Thus, zooplankton prey should be considered available to the examined CWCs. Accordingly, we hypothesize that the corals’ diet is most likely based on fresh phytodetritus, when available^100^, but zooplankton might contribute the most to the CWCs diet. However, the role of zooplankton as primary food source in the Angola CWC reefs still needs to be investigated in future studies.

Black coral and octocorals occupy the same trophic guild of *D. pertusum* and *M. oculata* (cluster 2, Fig. 1) however, their δ^13^C and δ^15^N signatures are more enriched than those of the latter two. These differences might be explained by taxon-specific (morphological and physiological) requirements^36,101^, since colony morphology^102^ and polyp size^103^ influence the rates of food capture^104^ and the selection of prey type and size^61^ between CWC species. This possibly leads to a less-opportunistic feeding strategy and food selection^61^ focused in capturing more of one type of food source, potentially zooplankton, resulting in differences in their trophic strategies^93^ and, consequently, isotopic signatures.

The crustacean decapod *Munida* sp., a fish of the family Myctophidae, and a polynoid polychaete also belong to the same trophic group as CWCs. This result highlights their suspension-feeding behavior and, most likely, all of them exploit the same food sources as the CWCs. Moreover, given that small fish are known to preferably feed on zooplankton^97^, this finding further supports the hypothesis of the importance of zooplankton as a food source to cluster 2. On the other hand, the polychaete *E. norvegica* and the analyzed echinoderms (Asteroids, Ophiuroids and *Echinus* sp.) are the most ^13^C-enriched organisms (δ^13^C from − 15.39 to − 12.81 ‰) with a corresponding mean TP of 4.16 ± 0.47 (cluster 3). The polychaete *E. norvegica* is known to live in close association with *D. pertusum* and *M. oculata*^13,105–107^ and to contribute to reef formation^106^, where the polychaete could benefit from extra food supply from the coral host by feeding directly on detritus in the corallites^105^. In the Angolan CWC reefs, *E. norvegica* was present at the basal part of the reef-building coral colonies^29^. The δ^15^N of *E. norvegica* is in the same range as the one for the other polychaete analyzed in this study. However, the comparative δ^13^C enrichment of *E. norvegica* indicates an exploitation of different food sources that could be related to its association with *D. pertusum* since it has been shown that, in the presence of the coral, *E. norvegica* assimilates more carbon through selective feeding on bigger particles^108^. It is possible that bigger particles consumed by *E. norvegica* indicate fresher particles, with a higher content of chlorophyll-a, resulting in ^13^C-enrichment^109^.

**Figure 3:**
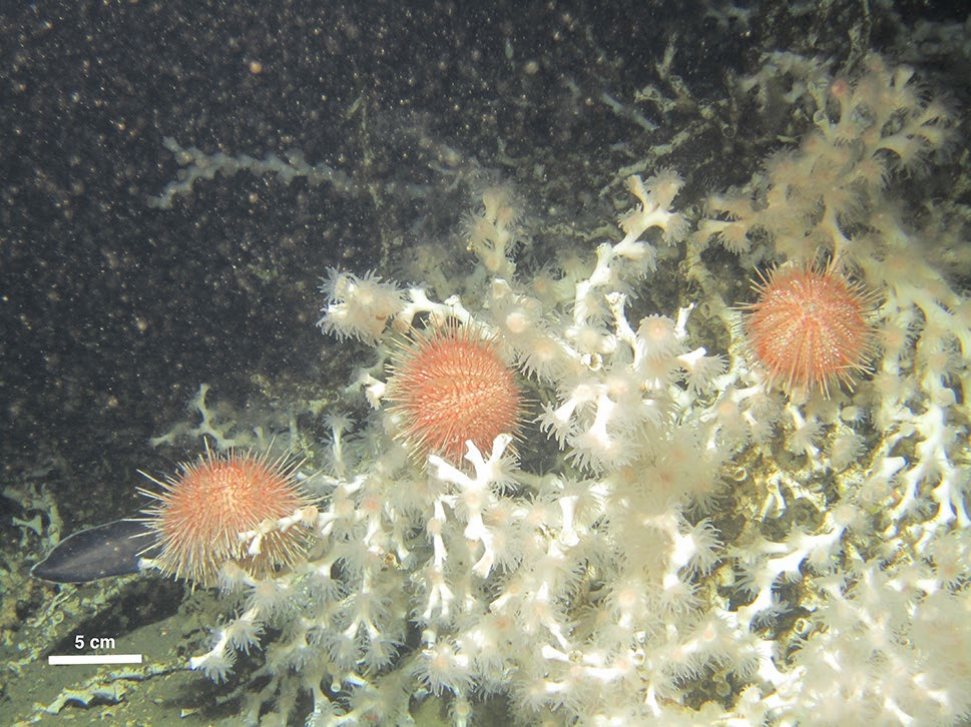
Sea urchins from the genus *Echinus* sp. on top of *Desmophyllum pertusum* at Valentine Mounds. Photo Credits: MARUM ROV SQUID.

The high standard deviation in δ^15^N of asteroids and in the δ^13^C of *Echinus* sp. could be attributed to the opportunistic feeding behavior of the organisms in these groups^110–113^. Given that all the organisms included in this trophic group are mobile species, they are potentially able to exploit different food sources within the reef, including detritus^31,114^, zooplankton^37,115^ or, by directly predating corals and sponges^116^. The latter seems more likely, since ROV video observations have shown the sea urchin *Echinus* sp. on top of *D. pertusum* colonies (Fig. 3), and the dissection of collected specimens confirmed the presence of fragments of *D. pertusum* in their gut’s contents^29^, supporting the evidence of its predatory feeding behavior. Moreover, echinoderms in the CWC reef presented the most enriched δ^13^C ratios (an enrichment of 5 ‰ compared to cluster 2). One possible explanation to the ^13^C-enrichement of this group could be a “deep-sea sponge loop”^40,70,117,118^, where the sponges’ feeding on bacteria and DOM, will result in the release of ^13^C-enriched particulate detritus that can be assimilated by associated fauna, both through detrital feeding and by directly feeding on sponges through a predatory pathway^40,118^. However, this interpretation should be taken with caution since in situ experiments are needed to support a “deep-sea sponge loop”.

Sponges are active filter-feeders and, therefore, it was expected that sponges act as primary consumers on the Angolan CWC reefs. However, sponges exhibited the highest δ^15^N values (from 17.33 to 21.90 ‰) of all samples analyzed in this study. These results are in the range of, and sometimes higher than, values usually attributed to top predators in marine trophic webs^119,120^. According to TP calculations following Post,^72^, the ^15^N-enriched ratios of sponges correspond to a mean TP of 5.69 ± 0.53 (Table 1), being inconsistent with the expected filter feeding behavior in the trophic web. Hanz et al.^70^ showed that TP calculations based on δ^15^N might not be appropriate to infer the TP of deep-sea sponges because when other biomarkers are used (such as compound specific isotope analyses), sponges act as normal filter feeders. Instead, the high δ^15^N values of sponges possibly indicate that sponges rely on additional food sources that have not been included in our stable isotope food model.

Sponges are capable of opportunistically exploiting a wide range of both organic and inorganic food sources, including DOM^45,121^, phyto- and bacterioplankton^118,122–125^, or even small zooplankton through the specialization of a group of sponges (Family Cladorhizidae) to utilize carnivory feeding modes^126,127^. In fact, the enriched δ^15^N values of hexactinellid sponges in the Angolan reefs are not entirely surprising since similarly high ratios have also been reported for deep-sea sponges in several other studies in the North Atlantic^37,79,98^, Pacific^125,128^, Arctic^70,114,129^ and Antarctic^66,130^ Oceans. The ^15^N-enriched values of sponges could be attributed to intense nitrogen cycling pathways in the sponge’s tissue^70,131^ driven by (1) the sponge holobiont^37,79^, defined here as the sponge and its associated microbial communities, and/or (2) the sponge’s feeding on heterotrophic re-suspended bacteria that are ^15^N-enriched because of the uptake of waste material of higher trophic levels^70,125,130^.

The Angolan CWC reefs occur in the center of the local OMZ and OMZs are zones with thriving microbial communities^55^ and complex nitrogen cycling dynamics^54,132,133^. The tissue of sponges is rich in microbial communities^53,129,134,135^ and the nitrogen isotope fractionation in sponges changes according to their bacterial richness^53,70,125^, i.e., if High Microbial Abundance (HMA) sponges or Low Microbial Abundance (LMA) sponges^70,136^. The sponge holobiont takes energy from DOM^137^ and, as a result of OM degradation, will release ammonium (NH_4_^+^)^70,123,138^, an isotopically depleted metabolic end-product^139^, leading to increased δ^15^N in the sponge tissue. Moreover, symbiotic relationships with bacteria, capable of both denitrification and anammox^131,140,141^**,** play an important role in sponge survival under low oxygen environmental conditions^52,53,142,143^. Likewise, it has been shown by Middelburg et al.^52^ that denitrification can occur in *D. pertusum*, due to the dominance of denitrifying bacteria in the coral’s holobiont and this process is enhanced by low-oxygen conditions. It is beyond the scope of our study to identify the exact physiological mechanisms used by benthic organisms to cope with low-oxygen environments, but the observed δ^15^N ratios of sponges in our study hints that interactions with microbes could be a plausible mechanism to withstand the hypoxic conditions. Given the slightly enriched δ^15^N values of *D. pertusum,* the CWC holobiont could also potentially play a role in the coral’s coping with the hypoxia conditions of the Angolan CWC reefs^50,52^, although this is more likely to be more significant for sponges given their enriched stable nitrogen isotope values.

This study gives the first insights into the trophic structure of the Angolan CWC reefs, where different feeding strategies are utilized by the organisms inhabiting in this OMZ. Our observations provide the first evidence pointing to an important role of zooplankton as food source for CWCs in Angola, and to a strong reliance of sponges, through their holobiont, on bacteria as food source in hypoxic conditions. The use of other biomarkers in future studies, such as compound specific stable isotope and fatty acids analyses, or in situ experiments with isotopically labelled food will contribute to a better understanding of CWCs and sponge’s nutrition. We further suggest that, in the future, special attention should be given to the potential role of the holobiont in allowing CWCs and sponges to withstand the conditions of the Angolan and other OMZs.

## Methods

### Study area

The Angola basin is located along the SW African continent in the SE Atlantic Ocean and belongs to the Benguela Current Large Marine Ecosystem (BCLME)^144^. The region is influenced by the Benguela upwelling system, one of the world’s most productive^145^, with an estimated primary production of 156 million t C yr^–1^^146^. Induced by coastal parallel winds, the upwelling of nutrient-rich cold waters results in enhanced primary productivity^81^, while the remineralization of high concentrations of OM in the water column results in severe mid-depth oxygen depletion and in a pronounced OMZ^147^ that coincides with the presence of the Angola CWC mounds^25^. In addition, the area receives a high input of riverine discharges from the Congo and Cuanza Rivers, which leads to further increase in primary production due to a high terrigenous nutrient input^11,82^. At approximately 17°S, the cold nutrient-rich waters transported northward by the Benguela Current interact with the warm nutrient-poor Angola Current^148^, forming the Angola-Benguela Frontal Zone (ABFZ)^149^. Oxygen-depleted and nutrient-rich waters are characteristic from 70 to 600 m water depth, indicating the presence of the South Atlantic Central Water (SACW)^150^, which is transported southward by the Angola Current.

The Angola CWC reefs cover long ridges to individual coral mounds that can reach up to 100 m above the surrounding sea floor. These CWC ridges and mounds have formed on millennial and longer time scales^151^, through successive periods of CWC reef development. They are composed of CWC fragments and other shells loosely embedded in fine hemipelagic sediments^25,29,30^.

### Sample collection

**Figure 4:**
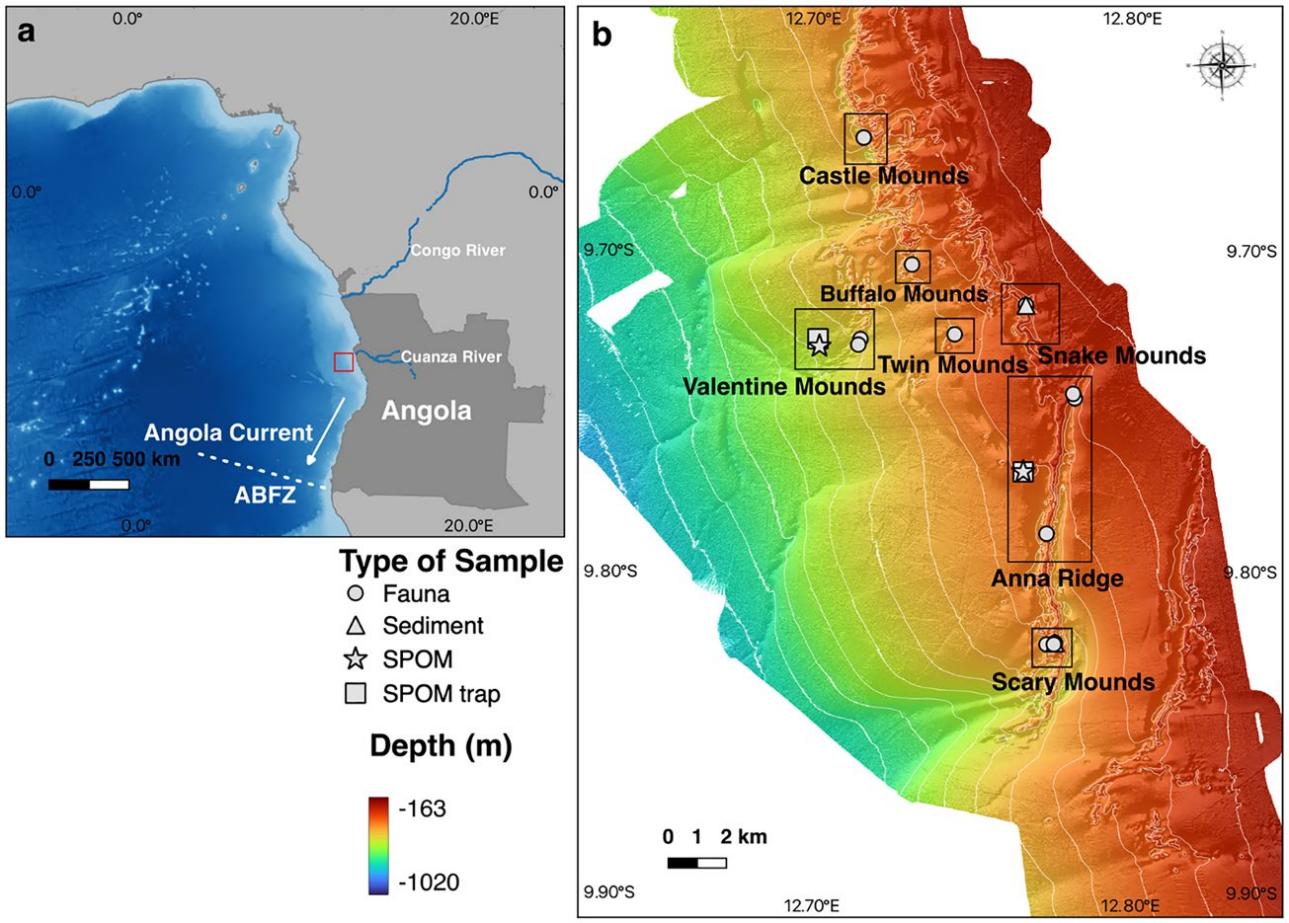
Study area map. (**a**) Location of the cold-water coral (CWC) mounds off Angola (red box) and of the Angola Current and the Angola-Benguela Frontal Zone (ABFZ). Bathymetry grid from GEBCO compilation^160^. (**b**) Locations, where different types of samples for stable isotope analysis were collected on the various CWC reefs (black boxes). Contour lines represent 50 m depth intervals. Bathymetry data was acquired during R/V Meteor cruise M122^161^. Maps created using the Open-Source software QGIS Version 3.20.3-Odense (http://www.qgis.org).

Samples were collected in January 2016 during the M122 (“ANNA”) expedition on board the R/V Meteor^29^. In total, 18 reef sites topping seven different CWC mound complexes (Fig. 4, Table 2) were sampled for stable isotope analyses. Samples of organisms belonging to the taxa Porifera, Cnidaria, Arthropoda, Annelida, Echinodermata and Chordata were collected by means of a box corer (box dimensions: 50 × 50 cm, 55 cm high), a Van-Veen grab sampler or the Remotely Operated Vehicle (ROV) SQUID (MARUM, Bremen, Germany)^29^ (Supplementary Table S1). Each organism was identified to the lowest possible taxonomic level, rinsed in seawater and put in plastic bags or vials.

**Table 2:** Metainformation of the number of samples collected (n), type of sample (Fauna, Sediment or suspended particulate organic matter (SPOM)) and sampling gear at the different sampling stations (Station ID).

Station ID	CWC mound	Latitude (S)	Longitude (E)	Depth (m)	n	Type of sample	Sampling method
GeoB 20913-1	Anna Ridge	9°47.296′	12°46.401′	307	1	Fauna	Grab sampler
GeoB 20920-1	Anna Ridge	9°44.763′	12°46.929′	336	8	Fauna	ROV
GeoB 20955-3	Anna Ridge	9°44.680′	12°46.896′	299	1	Fauna	Grab sampler
GeoB 20921-1	Anna Ridge	9°46.140′	12°45.960′	342	2	SPOM	Sediment trap
GeoB 20921-1	Anna Ridge	9°46.140′	12°45.960′	342	24	SPOM	McLane pump
GeoB 20927-1	Buffalo Mounds	9°42.270′	12°43.858′	402	10	Fauna	ROV
GeoB 20957	Castle Mounds	9°39.901′	12°42.945′	418	1	Fauna	ROV
GeoB 20957-1	Castle Mounds	9°39.901′	12°42.945′	447	2	Fauna	ROV
GeoB 20930-1	Scary Mounds	9°49.364′	12°46.405′	412	10	Fauna	ROV
GeoB 209332	Scary Mounds	9°49.336′	12°46.565′	345	1	Sediment	Grab sampler
GeoB 20933-3	Scary Mounds	9°49.337′	12°46.564′	346	2	Fauna	Grab sampler
GeoB 20934-2	Scary Mounds	9°49.362′	12°46.543′	382	1	Fauna	Grab sampler
GeoB 20953-2	Snake Mounds	9°43.026′	12°46.005′	259	3	Fauna and sediment	Box corer
GeoB 20910-1	Twin Mounds	9°43.573′	12°44.664′	334	1	Fauna	Grab sampler
GeoB 20917-1	Valentine Mounds	9°43.665′	12°42.891′	473	3	Fauna	ROV
GeoB 20904-1	Valentine Mounds	9°43.769′	12°42.851′	503	5	Fauna	ROV
GeoB 20916-1	Valentine Mounds	9°43.660′	12°42.090′	526	20	SPOM	Sediment trap
GeoB 20940-1	Valentine Mounds	9°43.800′	12°42.120′	532	24	SPOM	McLane pump

To investigate potential food sources for the megabenthic fauna, three types of POM were collected: SPOM from the water column, settling material from the sediment trap (SPOM trap), and sediment. SPOM was collected at two depths (342 and 532 m) at 40 cm above the seafloor using a McLane phytoplankton pump, attached to the ALBEX lander (NIOZ), with a maximum of 7.5 L of water pumped every 2 h for a period of 48 h over 24 GF/F filters (47 mm Whatman™ GF/F filters pre-combusted at 450 °C) (for more details see Hanz et al.^11^). Settling SPOM (SPOM trap) was also collected with a sediment trap (Technicap PPS4/3) attached to the ALBEX lander, with the collector at 2 m above the bottom over a sampling duration of 2.5 days, at both 342 and 526 m depth. Additionally, two sediment samples were collected at two depths (259 and 345 m): one with the box corer and another one with the Van-Veen grab sampler. All organisms and POM samples were stored on board at – 20 °C and, afterwards, were freeze-dried in the laboratory and stored at − 20 °C until further analysis.

### Sample preparation for stable isotope analyses

Fragments of freeze-dried faunal samples were ground to powder with a mortar and, depending on the quantity of sample available, two subsamples of 10 to 50 mg were weighted. One subsample was left untreated for δ^15^N determination, whereas the other was acidified with the addition of 10% HCl drop-by-drop until effervescence ceased and used for δ^13^C determination. The acidification of samples is a common practice in stable isotope analyses and aims to remove inorganic calcium carbonate from the organisms, known to interfere with δ^13^C ratios^112^. All samples analyzed for δ^13^C were acidified, except fish samples due to their low carbonate content^152^. After acidification, the subsamples were oven-dried at 50 °C for 72 h. Afterwards, three pseudo replicates of each subsample were weighted with a precision balance (± 0.001 mg) into tin capsules (11 × 4 mm, Elementar Microanalysis) to conduct the isotopic composition analyses. Depending on the taxonomic group, subsamples of 0.4 to 4 mg were weighted.

Sample preparation for the determination of δ^15^N and δ^13^C for the three types of POM considered (filtered SPOM, SPOM trap and sediment) is described in detail in Hanz et al.^9^. These samples were acidified by fuming HCl (20%) vapor over night for δ^13^C determination. Three replicates of sediment (1.6 to 3.6 mg for δ^13^C and 9 mg for δ^15^N) and sediment trap material (1 mg δ^13^C and 4 to 5 mg for δ^15^N) were weighted into tin capsules. For SPOM, a ¼ section of each GF/F filter was transferred into tin capsules for δ^13^C and δ^15^N analyses.

### Stable isotope analyses

Analyses of benthic megafauna samples were performed using the Elementar IsoPrime 100 isotope ratio–mass spectrometry (IR–MS) instrument (IsoPrime Ltd.) coupled to a CNS elemental analyzer (Elementar Vario Pyro Cube EA CNS; Elementar Analysensysteme GmbH). Stable carbon isotopic values (δ^13^C) were quality checked and calibrated by using the reference materials Glucose (BCR-657) and Polyethylene (IAEA-CH-7), while for the stable nitrogen isotopic (δ^15^N) values potassium nitrate (USGS32), caffeine (IAEA600) and ammonium sulfate (USGS25) were used.

The δ^15^N and δ^13^C values of filtered SPOM, SPOM trap and sediment were analyzed by a Delta V Advantage IR–MS coupled online to an elemental analyzer (Flash 2000 EA-IRMS) by a ConFlo IV (Thermo Fisher Scientific Inc.). Benzoic acid and acetanilide were used as standards for δ^13^C, whereas acetanilide, urea and casein were used as standards for δ^15^N (for more details see Hanz et al.^9^).

Vienna Pee Dee belemnite (V.P.D.B.) for carbon, and atmospheric N_2_ (Air) for nitrogen, were used as reference materials, and stable isotope values are here reported with respect to those. Precision is based on the standard deviation between replicate analyses and was < 0.50 ‰ for δ^15^N and < 0.20 ‰ for δ^13^C for faunal samples, and < 0.15 ‰ for δ^15^N and δ^13^C for POM.

Stable isotope ratios (δ^13^C and δ^15^N), in relation to the standards, were calculated as following:

1 $${\updelta }^{13}\mathrm{C or }{\updelta }^{15}\mathrm{N }(\mathrm{\permil }) = [\frac{\mathrm{R sample}}{\mathrm{R standard}}-1] \times 1000$$

where R corresponds to ^13^C/^12^C or ^15^N/^14^N of the analyzed sample (R_sample_) and standard used (R_standard_).

### Data analysis

The Trophic Position (TP) of consumers was calculated following Post^72^, as in similar studies on deep-sea habitats^98,115,153,154^. The TP of each consumer was calculated using the following equation:

2 $${\text{TP}}_{{\text{n}}} = \lambda + \left( {\delta^{{{15}}} {\text{N}}_{{\text{n}}} - \delta^{{{15}}} {\text{N}}_{{{\text{baseline}}}} } \right)/{\text{TEF}}$$

where TP_n_ corresponds to the trophic position of the organism, δ^15^N_n_ corresponds to its stable nitrogen isotope ratios, δ^15^N_baseline_ represents the nitrogen isotopic composition of the baseline, TEF corresponds to the Trophic Enrichment Factor for δ^15^N and λ represents the trophic level occupied by the baseline. The δ^15^N of the baseline considered was 4.23 ± 1.65 ‰, corresponding to the mean ratio measured for SPOM (λ = 1). A TEF of 3.4 ‰ for δ^15^N was considered, since it has been regarded as appropriate in other CWC habitats^31,37^.

To explore the different trophic guilds of the Angolan CWC reefs, a k-means clustering analysis was applied to the δ^13^C and δ^15^N data, computed using the “factoextra” package^155^ within R Studio^156^ under R version 4.1.3^157^. The optimal number of clusters (k = 4) was investigated using the “NbClust” R package^158^ by applying three different methods: elbow, silhouette, and gap statistic methods (Supplementary Fig. S2). The Pearson correlation between δ^13^C and δ^15^N ratios was calculated using the “cor.test” function available in the “stats” package in R. Since fauna and POM samples were collected along different CWC mounds, it was not possible in our study to assess how the stable isotopic ratios would differ between mounds.

## Supplementary Information

Supplementary Information.

## Data Availability

Dataset with samples metadata and carbon and nitrogen stable isotope values are available in Pangaea^159^ (https://doi.org/10.1594/PANGAEA.955091).

## References

[1] Mueller CE, Larsson AI, Veuger B, Middelburg JJ, van Oevelen D (2014). Opportunistic feeding on various organic food sources by the cold-water coral *Lophelia pertusa*. Biogeosciences.

[2] van Oevelen D (2009). The cold-water coral community as hotspot of carbon cycling on continental margins: A food-web analysis from Rockall Bank (northeast Atlantic). Limnol. Oceanogr..

[3] Cathalot C (2015). Cold-water coral reefs and adjacent sponge grounds: Hotspots of benthic respiration and organic carbon cycling in the deep sea. Front. Mar. Sci..

[4] Thiem Ø, Ravagnan E, Fosså JH, Berntsen J (2006). Food supply mechanisms for cold-water corals along a continental shelf edge. J. Mar. Syst..

[5] Mohn C (2014). Linking benthic hydrodynamics and cold-water coral occurrences: A high-resolution model study at three cold-water coral provinces in the NE Atlantic. Prog. Oceanogr..

[6] Frederiksen R, Jensen A, Westerberg H (1992). The distribution of the scleractinian coral *Lophelia pertusa* around the Faroe islands and the relation to internal tidal mixing. Sarsia.

[7] van Haren H, Mienis F, Duineveld GCA, Lavaleye MSS (2014). High-resolution temperature observations of a trapped nonlinear diurnal tide influencing cold-water corals on the Logachev mounds. Prog. Oceanogr..

[8] Davies AJ (2009). Downwelling and deep-water bottom currents as food supply mechanisms to the cold-water coral *Lophelia pertusa* (Scleractinia) at the Mingulay Reef Complex. Limnol. Oceanogr..

[9] Hebbeln D (2014). Environmental forcing of the Campeche cold-water coral province, southern Gulf of Mexico. Biogeosciences.

[10] de Clippele LH (2017). Using novel acoustic and visual mapping tools to predict the small-scale spatial distribution of live biogenic reef framework in cold-water coral habitats. Coral Reefs.

[11] Hanz U (2019). Environmental factors influencing benthic communities in the oxygen minimum zones on the Angolan and Namibian margins. Biogeosciences.

[12] Addamo AM (2016). Merging scleractinian genera: The overwhelming genetic similarity between solitary *Desmophyllum* and colonial *Lophelia*. BMC Evol. Biol..

[13] Buhl-Mortensen L (2010). Biological structures as a source of habitat heterogeneity and biodiversity on the deep ocean margins. Mar. Ecol..

[14] Price DM, Robert K, Callaway A, Hall RA, Huvenne VAI (2019). Using 3D photogrammetry from ROV video to quantify cold-water coral reef structural complexity and investigate its influence on biodiversity and community assemblage. Coral Reefs.

[15] Hennige SJ (2021). Using the Goldilocks Principle to model coral ecosystem engineering. Proc. R. Soc. B.

[16] Henry LA, Roberts JM (2007). Biodiversity and ecological composition of macrobenthos on cold-water coral mounds and adjacent off-mound habitat in the bathyal Porcupine Seabight, NE Atlantic. Deep Sea Res. Part I.

[17] Lessard-Pilon SA, Podowski EL, Cordes EE, Fisher CR (2010). Megafauna community composition associated with *Lophelia pertusa* colonies in the Gulf of Mexico. Deep Sea Res. Part II.

[18] Rueda JL (2019). Cold-water coral associated fauna in the Mediterranean Sea and adjacent areas. Mediterr. Cold-Water Corals.

[19] Costello MJ (2005). Role of cold-water coral reefs as fish habitat in the NE Atlantic. Cold-Water Corals Ecosyst..

[20] Purser A (2013). Local variation in the distribution of benthic megafauna species associated with cold-water coral reefs on the Norwegian margin. Cont. Shelf Res..

[21] Buhl-Mortensen P, Hovland M, Brattegard T, Farestveit R (1995). Deep water bioherms of the scleractinian coral *Lophelia*
*pertusa* (L) at 64° N on the Norwegian shelf: Structure and associated megafauna. Sarsia.

[22] Buhl-Mortensen P, Buhl-Mortensen L, Purser A, Rossi S, Bramanti L, Gori A, Orejas C (2017). Trophic ecology and habitat provision in cold-water coral ecosystems. Marine Animal Forests.

[23] Bartzke G (2021). Investigating the prevailing hydrodynamics around a cold-water coral colony using a physical and a numerical approach. Front. Mar. Sci..

[24] Corbera G (2022). Local-scale feedbacks influencing cold-water coral growth and subsequent reef formation. Sci. Rep..

[25] Hebbeln D (2020). Cold-water coral reefs thriving under hypoxia. Coral Reefs.

[26] Orejas C (2021). *Madrepora*
*oculata* forms large frameworks in hypoxic waters off Angola (SE Atlantic). Sci. Rep..

[27] Dodds LA, Roberts JM, Taylor AC, Marubini F (2007). Metabolic tolerance of the cold-water coral *Lophelia pertusa* (Scleractinia) to temperature and dissolved oxygen change. J. Exp. Mar. Biol. Ecol..

[28] Davies AJ, Wisshak M, Orr JC, Murray Roberts J (2008). Predicting suitable habitat for the cold-water coral *Lophelia*
*pertusa* (Scleractinia). Deep Sea Res. Part I.

[29] Hebbeln, D. *et al.* ANNA Cold-Water Coral Ecosystems off Angola and Namibia, Cruise No. M122, 30 December 2015–31 January 2016, Walvis Bay (Namibia), Walvis Bay (Namibia). *METEOR-Berichte.*10.2312/cr_m122 (2017).

[30] Wienberg, C. *et al.* (accepted) Cold-water coral reefs in the oxygen minimum zones off West Africa. In *Cold-Water Coral Reefs of the World* (eds. Cordes, E. & Mienis, F. ) (Springer series: Coral Reefs of the World).

[31] Carlier A (2009). Trophic relationships in a deep Mediterranean cold-water coral bank (Santa Maria di Leuca, Ionian Sea). Mar. Ecol. Prog. Ser..

[32] Gori A (2011). Size and spatial structure in deep versus shallow populations of the Mediterranean gorgonian *Eunicella*
*singularis* (Cap de Creus, northwestern Mediterranean Sea). Mar. Biol..

[33] Coppari M (2020). Seasonal variation of the stable C and N isotopic composition of the mesophotic black coral *Antipathella*
*subpinnata* (Ellis & Solander, 1786). Estuar Coast Shelf Sci..

[34] Duineveld GCA, Lavaleye MSS, Berghuis EM (2004). Particle flux and food supply to a seamount cold-water coral community (Galicia Bank, NW Spain). Mar. Ecol. Prog. Ser..

[35] Duineveld GCA, Lavaleye MSS, Bergman MJN, de Stigter H, Mienis F (2007). Trophic structure of a cold-water coral mound community (Rockall Bank, NE Atlantic) in relation to the near-bottom particle supply and current regime. Bull. Mar. Sci..

[36] Sherwood OA, Jamieson RE, Edinger EN, Wareham VE (2008). Stable C and N isotopic composition of cold-water corals from the Newfoundland and Labrador continental slope: Examination of trophic, depth and spatial effects. Deep Sea Res. Part I.

[37] van Oevelen D, Duineveld GCA, Lavaleye MSS, Kutti T, Soetaert K (2018). Trophic structure of cold-water coral communities revealed from the analysis of tissue isotopes and fatty acid composition. Mar. Biol. Res..

[38] Becker EL, Cordes EE, Macko SA, Fisher CR (2009). Importance of seep primary production to *Lophelia*
*pertusa* and associated fauna in the Gulf of Mexico. Deep Sea Res. Part I.

[39] Gori A, Grover R, Orejas C, Sikorski S, Ferrier-Pagès C (2014). Uptake of dissolved free amino acids by four cold-water coral species from the Mediterranean Sea. Deep Sea Res. Part II.

[40] Maier SR (2020). Recycling pathways in cold-water coral reefs: Use of dissolved organic matter and bacteria by key suspension feeding taxa. Sci. Rep..

[41] Duineveld GCA (2012). Spatial and tidal variation in food supply to shallow cold-water coral reefs of the Mingulay Reef complex (Outer Hebrides, Scotland). Mar. Ecol. Prog. Ser..

[42] Dodds LA, Black KD, Orr H, Roberts JM (2009). Lipid biomarkers reveal geographical differences in food supply to the cold-water coral *Lophelia*
*pertusa* (Scleractinia). Mar. Ecol. Prog. Ser..

[43] Naumann MS, Orejas C, Wild C, Ferrier-Pagès C (2011). First evidence for zooplankton feeding sustaining key physiological processes in a scleractinian cold-water coral. J. Exp. Biol..

[44] Kiriakoulakis K (2005). Lipids and nitrogen isotopes of two deep-water corals from the North-East Atlantic: Initial results and implications for their nutrition. Cold-Water Corals and Ecosystems.

[45] Maier SR, Bannister RJ, van Oevelen D, Kutti T (2020). Seasonal controls on the diet, metabolic activity, tissue reserves and growth of the cold-water coral *Lophelia*
*pertusa*. Coral Reefs.

[46] Messié M, Chavez FP (2015). Seasonal regulation of primary production in eastern boundary upwelling systems. Prog. Oceanogr..

[47] Levin LA, Huggett CL, Wishner KF (1991). Control of deep-sea benthic community structure by oxygen and organic-matter gradients in the eastern Pacific Ocean. J. Mar. Res..

[48] Diaz RJ, Rosenberg R (1995). Marine benthic hypoxia: A review of its ecological effects and the behavioural responses of benthic macrofauna. Oceanogr. Mar. Biol..

[49] Levin LA (2003). Oxygen minimum zone benthos: Adaptation and community response to hypoxia. Oceanogr. Mar. Biol. Annu. Rev..

[50] Gori A (2023). Natural hypoxic conditions do not affect the respiration rates of the cold-water coral *Desmophyllum*
*pertusum* (*Lophelia*
*pertusa*) living in the Angola margin (Southeastern Atlantic Ocean). Deep Sea Res. Part I.

[51] Hoffmann F (2007). An anaerobic world in sponges. Geomicrobiol. J..

[52] Middelburg JJ (2015). Discovery of symbiotic nitrogen fixation and chemoautotrophy in cold-water corals. Sci. Rep..

[53] Maldonado M (2021). A microbial nitrogen engine modulated by Bacteriosyncytia in hexactinellid sponges: Ecological implications for deep-sea communities. Front. Mar. Sci..

[54] Lam P, Kuypers MMM (2010). Microbial nitrogen cycling processes in oxygen minimum zones. Ann. Rev. Mar. Sci..

[55] Wright JJ, Konwar KM, Hallam SJ (2012). Microbial ecology of expanding oxygen minimum zones. Nat. Rev. Microbiol..

[56] Levin LA (2009). Effects of natural and human-induced hypoxia on coastal benthos. Biogeosciences.

[57] Kalvelage T (2015). Aerobic microbial respiration in oceanic oxygen minimum zones. PLoS ONE.

[58] Churchill DA (2015). Trophic interactions of common elasmobranchs in deep-sea communities of the Gulf of Mexico revealed through stable isotope and stomach content analysis. Deep Sea Res. Part II.

[59] Hoving HJT, Haddock SHD (2017). The giant deep-sea octopus *Haliphron*
*atlanticus* forages on gelatinous fauna. Sci. Rep..

[60] Coma R, Gili J-M, Zabala M, Riera T (1994). Feeding and prey capture cycles in the aposymbiontic gorgonian *Paramuricea*
*clavata*. Mar. Ecol. Prog. Ser..

[61] Orejas C, Gili JM, Arntz W (2003). The role of the small planktonic communities in the diet of two Antarctic octocorals (*Primnoisis antarctica* and *Primnoella* sp.). Mar. Ecol. Prog. Ser..

[62] Hoving HJT, Robison BH (2016). Deep-sea in situ observations of gonatid squid and their prey reveal high occurrence of cannibalism. Deep Sea Res. Part I.

[63] Choy CA, Haddock SHD, Robison BH (2017). Deep pelagic food web structure as revealed by in situ feeding observations. Proc. R. Soc. B.

[64] Iken K, Bluhm BA, Gradinger R (2005). Food web structure in the high Arctic Canada Basin: Evidence from δ^13^C and δ^15^N analysis. Polar Biol..

[65] Polunin NVC (2001). Feeding relationships in Mediterranean bathyal assemblages elucidated by stable nitrogen and carbon isotope data. Mar. Ecol. Prog. Ser..

[66] Mincks SL, Smith CR, Jeffreys RM, Sumida PYG (2008). Trophic structure on the West Antarctic Peninsula shelf: Detritivory and benthic inertia revealed by δ^13^C and δ^15^N analysis. Deep Sea Res. Part II.

[67] Boyle MD, Ebert DA, Cailliet GM (2012). Stable-isotope analysis of a deep-sea benthic-fish assemblage: Evidence of an enriched benthic food web. J. Fish Biol..

[68] Rossi S, Elias-Piera F (2018). Trophic ecology of three echinoderms in deep waters of the Weddell Sea (Antarctica). Mar. Ecol. Prog. Ser..

[69] Puccinelli E, Smart SM, Fawcett SE (2020). Temporal variability in the trophic composition of benthic invertebrates in the Indian Sub-Antarctic Ocean. Deep Sea Res. Part I.

[70] Hanz U (2022). The important role of sponges in carbon and nitrogen cycling in a deep-sea biological hotspot. Funct. Ecol..

[71] Tieszen LL, Boutton TW, Tesdahl KG, Slade NA (1983). Fractionation and turnover of stable carbon isotopes in animal tissues: Implications for δ^13^C analysis of diet. Oecologia.

[72] Post DM (2002). Using stable isotopes to estimate trophic position: Models, methods, and assumptions. Ecology.

[73] Deniro MJ, Epstein S (1981). Influence of diet on the distribution of nitrogen isotopes in animals. Geochim. Cosmochim. Acta.

[74] Fry B, Sherr EB (1989). δC Measurements as Indicators of Carbon Flow in Marine and Freshwater Ecosystems.

[75] Smith BN, Epstein S (1971). Two categories of ^13^C/^12^C ratios for higher plants. Plant Physiol..

[76] Yamanaka, T. *et al.* A compilation of the stable isotopic compositions of Carbon, Nitrogen, and Sulfur in soft body parts of animals collected from deep-sea hydrothermal vent and methane seep fields: Variations in energy source and importance of subsurface microbial processes in the sediment-hosted systems. In *Subseafloor biosphere linked to hydrothermal systems* 105–129 (Springer, 2015). 10.1007/978-4-431-54865-2_10.

[77] Sweetman AK (2017). Major impacts of climate change on deep-sea benthic ecosystems. Elementa.

[78] le Guilloux E (2009). First observations of deep-sea coral reefs along the Angola margin. Deep Sea Res. Part II.

[79] Iken K, Brey T, Wand U, Voigt J, Junghans P (2001). Food web structure of the benthic community at the Porcupine Abyssal Plain (NE Atlantic): A stable isotope analysis. Prog. Oceanogr..

[80] Fanelli E, Cartes JE, Papiol V (2011). Food web structure of deep-sea macrozooplankton and micronekton off the Catalan slope: Insight from stable isotopes. J. Mar. Syst..

[81] Carr ME, Kearns EJ (2003). Production regimes in four Eastern Boundary Current systems. Deep Sea Res. Part II.

[82] Baudin F (2017). Organic carbon accumulation in modern sediments of the Angola basin influenced by the Congo deep-sea fan. Deep Sea Res. Part II.

[83] Juva K, Flögel S, Karstensen J, Linke P, Dullo WC (2020). Tidal dynamics control on cold-water coral growth: A high-resolution multivariable study on Eastern Atlantic cold-water coral sites. Front. Mar. Sci..

[84] da Portilho-Ramos RC (2022). Major environmental drivers determining life and death of cold-water corals through time. PLoS Biol..

[85] Mienis F (2012). The influence of near-bed hydrodynamic conditions on cold-water corals in the Viosca Knoll area, Gulf of Mexico. Deep Sea Res. Part I.

[86] Signorini SR (1999). Biological and physical signatures in the tropical and subtropical Atlantic. J. Geophys. Res. Oceans.

[87] Reason CJC, Landman W, Tennant W (2006). Seasonal to decadal prediction of Southern African climate and its links with variability of the Atlantic Ocean. Bull. Am. Meteorol. Soc..

[88] Gori A (2012). Reproductive cycle and trophic ecology in deep versus shallow populations of the Mediterranean gorgonian *Eunicella*
*singularis* (Cap de Creus, northwestern Mediterranean Sea). Coral Reefs.

[89] de Froe E (2022). Hydrography and food distribution during a tidal cycle above a cold-water coral mound. Deep Sea Res. Part I.

[90] Kiriakoulakis K, Bett BJ, White M, Wolff GA (2004). Organic biogeochemistry of the Darwin Mounds, a deep-water coral ecosystem, of the NE Atlantic. Deep Sea Res. Part I.

[91] Naumann MS, Tolosa I, Taviani M, Grover R, Ferrier-Pagès C (2015). Trophic ecology of two cold-water coral species from the Mediterranean Sea revealed by lipid biomarkers and compound-specific isotope analyses. Coral Reefs.

[92] Sherwood OA (2005). Stable isotopic composition of deep-sea gorgonian corals *Primnoa* spp.: A new archive of surface processes. Mar. Ecol. Prog. Ser..

[93] Rakka M (2021). Contrasting metabolic strategies of two co-occurring deep-sea octocorals. Sci. Rep..

[94] Rakka M (2020). Feeding biology of a habitat-forming antipatharian in the Azores Archipelago. Coral Reefs.

[95] Teuber L (2013). Distribution and ecophysiology of calanoid copepods in relation to the oxygen minimum zone in the eastern tropical Atlantic. PLoS ONE.

[96] Postel L (2007). Zooplankton biomass variability off Angola and Namibia investigated by a lowered ADCP and net sampling. J. Mar. Syst..

[97] Bode A (2003). The pelagic foodweb in the upwelling ecosystem of Galicia (NW Spain) during spring: Natural abundance of stable carbon and nitrogen isotopes. ICES J. Mar. Sci..

[98] Colaço A, Giacomello E, Porteiro F, Menezes GM (2013). Trophodynamic studies on the condor seamount (Azores, Portugal, North Atlantic). Deep Sea Res. Part II.

[99] Figueiredo GGAA (2020). Body size and stable isotope composition of zooplankton in the western tropical Atlantic. J. Mar. Syst..

[100] Gori A (2018). Biochemical composition of the cold-water coral Dendrophyllia cornigera under contrasting productivity regimes: Insights from lipid biomarkers and compound-specific isotopes. Deep Sea Res. Part I.

[101] Salvo F, Hamoutene D, Hayes VEW, Edinger EN, Parrish CC (2018). Investigation of trophic ecology in Newfoundland cold-water deep-sea corals using lipid class and fatty acid analyses. Coral Reefs.

[102] Mortensen PB, Buhl-Mortensen L (2005). Morphology and growth of the deep-water gorgonians *Primnoa*
*resedaeformis* and *Paragorgia*
*arborea*. Mar. Biol..

[103] Palardy JE, Grottoli AG, Matthews KA (2005). Effects of upwelling, depth, morphology and polyp size on feeding in three species of *Panamanian* corals. Mar. Ecol. Prog. Ser..

[104] Tsounis G (2010). Prey-capture rates in four Mediterranean cold water corals. Mar. Ecol. Prog. Ser..

[105] Mortensen PB (2001). Aquarium observations on the deep-water coral *Lophelia*
*pertusa* (L, 1758) (scleractinia) and selected associated invertebrates. Ophelia.

[106] Roberts JM (2005). Reef-aggregating behaviour by symbiotic eunicid polychaetes from cold-water corals: Do worms assemble reefs?. J. Mar. Biol. Assoc. U.K..

[107] Oppelt A, López Correa M, Rocha C (2017). Biogeochemical analysis of the calcification patterns of cold-water corals *Madrepora*
*oculata* and *Lophelia*
*pertusa* along contact surfaces with calcified tubes of the symbiotic polychaete *Eunice*
*norvegica*: Evaluation of a ‘mucus’ calcification hypothesis. Deep Sea Res. Part I.

[108] Mueller CE, Lundälv T, Middelburg JJ, van Oevelen D (2013). The symbiosis between *Lophelia*
*pertusa* and *Eunice*
*norvegica* stimulates coral calcification and worm assimilation. PLoS ONE.

[109] Miller RJ, Page HM, Brzezinski MA (2013). δ^13^C and δ^15^N of particulate organic matter in the Santa Barbara Channel: Drivers and implications for trophic inference. Mar. Ecol. Prog. Ser..

[110] Gale KSP, Hamel J-F, Mercier A (2013). Trophic ecology of deep-sea Asteroidea (Echinodermata) from eastern Canada. Deep Sea Res. Part I.

[111] Michel LN, David B, Dubois P, Lepoint G, de Ridder C (2016). Trophic plasticity of Antarctic echinoids under contrasted environmental conditions. Polar Biol..

[112] Jacob U, Mintenbeck K, Brey T, Knust R, Beyer K (2005). Stable isotope food web studies: A case for standardized sample treatment. Mar. Ecol. Prog. Ser..

[113] Jacob U (2006). Towards the trophic structure of the Bouvet Island marine ecosystem. Polar Biol..

[114] Bergmann M, Dannheim J, Bauerfeind E, Klages M (2009). Trophic relationships along a bathymetric gradient at the deep-sea observatory HAUSGARTEN. Deep Sea Res. Part I.

[115] Parzanini C, Parrish CC, Hamel J-F, Mercier A (2018). Trophic relationships of deep-sea benthic invertebrates on a continental margin in the NW Atlantic inferred by stable isotope, elemental, and fatty acid composition. Prog. Oceanogr..

[116] Bo M, Canese S, Bavestrello G (2019). On the coral-feeding habit of the sea star *Peltaster*
*placenta*. Mar. Biodivers..

[117] Rix L (2016). Coral mucus fuels the sponge loop in warm- and cold-water coral reef ecosystems. Sci. Rep..

[118] Bart MC, Hudspith M, Rapp HT, Verdonschot PFM, de Goeij JM (2021). A deep-sea sponge loop? Sponges transfer dissolved and particulate organic carbon and nitrogen to associated fauna. Front. Mar. Sci..

[119] Borisova EA (2020). Ecotype and geographical variation in carbon and nitrogen stable isotope values in western North Pacific killer whales (*Orcinus*
*orca*). Mar. Mamm. Sci..

[120] Pethybridge H (2018). A global meta-analysis of marine predator nitrogen stable isotopes: Relationships between trophic structure and environmental conditions. Glob. Ecol. Biogeogr..

[121] Bart MC (2020). Differential processing of dissolved and particulate organic matter by deep-sea sponges and their microbial symbionts. Sci. Rep..

[122] Pile AJ, Young CM (2006). The natural diet of a hexactinellid sponge: Benthic–pelagic coupling in a deep-sea microbial food web. Deep Sea Res. Part I.

[123] Yahel G, Whitney F, Reiswig HM, Eerkes-Medrano DI, Leys SP (2007). In situ feeding and metabolism of glass sponges (Hexactinellida, Porifera) studied in a deep temperate fjord with a remotely operated submersible. Limnol. Oceanogr..

[124] Robertson LM, Hamel JF, Mercier A (2017). Feeding in deep-sea demosponges: Influence of abiotic and biotic factors. Deep Sea Res. Part I.

[125] Kahn AS, Chu JWF, Leys SP (2018). Trophic ecology of glass sponge reefs in the Strait of Georgia, British Columbia. Sci. Rep..

[126] Vacelet J, Boury-Esnault N (1995). Carnivorous sponges. Nature.

[127] Godefroy N (2019). Sponge digestive system diversity and evolution: Filter feeding to carnivory. Cell Tissue Res..

[128] Archer SK (2020). Foundation species abundance influences food web topology on glass sponge reefs. Front. Mar. Sci..

[129] Morganti TM (2022). Giant sponge grounds of Central Arctic seamounts are associated with extinct seep life. Nat. Commun..

[130] Mintenbeck K, Jacob U, Knust R, Arntz WE, Brey T (2007). Depth-dependence in stable isotope ratio δ^15^N of benthic POM consumers: The role of particle dynamics and organism trophic guild. Deep Sea Res. Part I.

[131] Pita L, Rix L, Slaby BM, Franke A, Hentschel U (2018). The sponge holobiont in a changing ocean: From microbes to ecosystems. Microbiome.

[132] Kalvelage T (2011). Oxygen sensitivity of anammox and coupled N-cycle processes in oxygen minimum zones. PLoS ONE.

[133] Kalvelage T (2013). Nitrogen cycling driven by organic matter export in the South Pacific oxygen minimum zone. Nat. Geosci..

[134] de Kluijver A (2021). Bacterial precursors and unsaturated long-chain fatty acids are biomarkers of North-Atlantic deep-sea demosponges. PLoS ONE.

[135] Busch K (2022). Biodiversity, environmental drivers, and sustainability of the global deep-sea sponge microbiome. Nat. Commun..

[136] Weisz JB, Hentschel U, Lindquist N, Martens CS (2007). Linking abundance and diversity of sponge-associated microbial communities to metabolic differences in host sponges. Mar. Biol..

[137] de Kluijver A (2021). An integrative model of carbon and nitrogen metabolism in a common deep-sea sponge (*Geodia*
*barretti*). Front. Mar. Sci..

[138] Kahn AS, Yahel G, Chu JWF, Tunnicliffe V, Leys SP (2015). Benthic grazing and carbon sequestration by deep-water glass sponge reefs. Limnol. Oceanogr..

[139] Montoya JP (2008). Nitrogen stable isotopes in marine environments. Nitrogen Mar. Environ..

[140] Schläppy ML (2010). Evidence of nitrification and denitrification in high and low microbial abundance sponges. Mar. Biol..

[141] Hoffmann F (2009). Complex nitrogen cycling in the sponge *Geodia*
*barretti*. Environ. Microbiol..

[142] Rubin-Blum M (2019). Fueled by methane: Deep-sea sponges from asphalt seeps gain their nutrition from methane-oxidizing symbionts. ISME J..

[143] Schuster A, Strehlow BW, Eckford-Soper L, McAllen R, Canfield DE (2021). Effects of seasonal anoxia on the microbial community structure in demosponges in a marine lake in Lough Hyne, Ireland. mSphere.

[144] Kainge P (2020). Fisheries yields, climate change, and ecosystem-based management of the Benguela Current Large Marine Ecosystem. Environ. Dev..

[145] Messié M (2009). Potential new production estimates in four eastern boundary upwelling ecosystems. Prog. Oceanogr..

[146] Demarcq H, Richardson AJ, Field JG (2008). Generalised model of primary production in the southern Benguela upwelling system. Mar. Ecol. Prog. Ser..

[147] Chapman P, Shannon LV (1987). Seasonality in the oxygen minimum layers at the extremities of the Benguela system. S. Afr. J. Mar. Sci..

[148] Kopte R (2017). The Angola Current: Flow and hydrographic characteristics as observed at 11°S. J. Geophys. Res. Oceans.

[149] John HC (2004). Oceanographic and faunistic structures across an Angola Current intrusion into northern Namibian waters. J. Mar. Syst..

[150] Stramma L, England M (1999). On the water masses and mean circulation of the South Atlantic Ocean. J. Geophys. Res. Oceans.

[151] Wefing AM (2017). High precision U-series dating of scleractinian cold-water corals using an automated chromatographic U and Th extraction. Chem. Geol..

[152] Carabel S, Godínez-Domínguez E, Verísimo P, Fernández L, Freire J (2006). An assessment of sample processing methods for stable isotope analyses of marine food webs. J. Exp. Mar. Biol. Ecol..

[153] Nyssen F (2002). A stable isotope approach to the eastern Weddell Sea trophic web: Focus on benthic amphipods. Polar Biol..

[154] Quiroga E, Gerdes D, Montiel A, Knust R, Jacob U (2014). Normalized biomass size spectra in high Antarctic macrobenthic communities: Linking trophic position and body size. Mar. Ecol. Prog. Ser..

[155] Kassambara, A. & Mundt, F. Extract and visualize the results of multivariate data analyses. R Package Version 1.0. 5. *R package version* (2020).

[156] Team, Rs. *RStudio: Integrated Development for R*. (RStudio, Inc., 2022) http://www.rstudio.com.

[157] Team, R. C. *R: A Language and Environment for Statistical Computing*. (R Foundation for Statistical Computing, 2022). http://www.R-project.org/.

[158] Charrad M, Ghazzali N, Boiteau V, Niknafs A (2014). NbClust: An R package for determining the relevant number of clusters in a data set. J. Stat. Softw..

[159] Vinha, B. *et al.* Carbon and nitrogen stable isotopes of benthic megafauna on the angolan cold-water coral reefs (SE Atlantic). *PANGAEA.*10.1594/PANGAEA.955091 (2023).

[160] GEBCO Compilation Group. GEBCO_2022 Grid. (2022).

[161] Hebbeln, D. *et al.**Multibeam Bathymetry Raw Data (Kongsberg EM710 Entire Dataset) of RV METEOR During Cruise M122*. 10.1594/PANGAEA.945015 (2022).

