# Supplementary material for: Trophic ecology of Angolan cold-water coral reefs (SE Atlantic) based on stable isotope analyses
Source: Sci Rep. 2023 Jun 19;13:9933. doi: 10.1038/s41598-023-37035-x (PMC10279766; doi:10.1038/s41598-023-37035-x)

Trophic ecology of Angolan cold-water coral reefs (SE Atlantic) based on stable isotope analyses

Beatriz Vinha, Sergio Rossi, Andrea Gori, Ulrike Hanz, Antonio Pennetta, Giuseppe E. De Benedetto, Furu Mienis, Veerle A. I. Huvenne, Dierk Hebbeln, Claudia Wienberg, Jürgen Titschack, André Freiwald, Stefano Piraino, Covadonga Orejas

Supplementary Table S1. Metainformation of all the samples collected for stable isotopes analysis.

Station	Site	Latitude (S)	Longitude (E)	Depth (m)	Sampling gear	Taxon	Taxonomic Group	Label
GeoB 20934-2	Scary Mounds	9º49.362	12º46.543	382	Grab	Cnidaria	Paramuricea sp	Par
GeoB 20930-1	Scary Mounds	9º49.364	12º46.405	412	ROV	Cnidaria	Paramuricea sp	Par
GeoB 20927-1	Buffalo Mounds	9º42.270	12º43.858	402	ROV	Cnidaria	Paramuricea sp	Par
GeoB 20957	Castle Mounds	9º39.901	12º42.945	418	ROV	Cnidaria	Callogorgia sp	Cal
GeoB 20933-3	Scary Mounds	9º49.337	12º46.564	346	Grab	Cnidaria	Eunicella sp	Eun
GeoB 20927-1	Buffalo Mounds	9º42.270	12º43.858	402	ROV	Cnidaria	Eunicella sp	Eun
GeoB 20930-1	Scary Mounds	9º49.364	12º46.405	412	ROV	Cnidaria	Spinimuricea sp	Spi
GeoB 20957-1	Castle Mounds	9º39.901	12º42.945	447	ROV	Cnidaria	Gorgoniidae	Gorg
GeoB 20930-1	Scary Mounds	9º49.364	12º46.405	412	ROV	Cnidaria	Gorgoniidae	Gorg
GeoB 20930-1	Scary Mounds	9º49.364	12º46.405	412	ROV	Cnidaria	Gorgoniidae	Gorg
GeoB 20927-1	Buffalo Mounds	9º42.270	12º43.858	402	ROV	Cnidaria	Gorgoniidae	Gorg
GeoB 20927-1	Buffalo Mounds	9º42.270	12º43.858	402	ROV	Cnidaria	Clavularia sp	Clav
GeoB 20955-3	Anna Ridge	9º44.680	12º46.896	299	Grab	Cnidaria	Clavularia sp	Clav
GeoB 20930-1	Scary Mounds	9º49.364	12º46.405	412	ROV	Cnidaria	Clavularia sp	Clav
GeoB 20927-1	Buffalo Mounds	9º42.270	12º43.858	402	ROV	Cnidaria	Parantipathes sp	Parant
GeoB20927-1	Buffalo Mounds	9º42.270	12º43.858	402	ROV	Cnidaria	*Desmophyllum pertusum*	D_pert
GeoB20917-1	Valentine Mounds	9º43.769	12º42.851	473	ROV	Cnidaria	*Desmophyllum pertusum*	D_pert

GeoB20904-1	Valentine Mounds	9º43.769	12º42.851	502	ROV	Cnidaria	*Desmophyllum pertusum*	D_pert
GeoB20917-1	Valentine Mounds	9º43.769	12º42.851	473	ROV	Cnidaria	*Desmophyllum pertusum*	D_pert
GeoB20927-1	Buffalo Mounds	9º42.270	12º43.858	402	ROV	Cnidaria	*Madrepora oculata*	Madr
GeoB20930-1	Scary Mounds	9º49.364	12º46.405	425	ROV	Cnidaria	*Madrepora oculata*	Madr
GeoB 20933-3	Scary Mounds	9º49.337	12º46.564	346	Grab	Cnidaria	Actiniaria	Actin
GeoB 20930-1	Scary Mounds	9º49.364	12º46.405	412	ROV	Cnidaria	Actiniaria	Actin
GeoB 20927-1	Buffalo Mounds	9º42.270	12º43.858	402	ROV	Cnidaria	Actiniaria	Actin
GeoB 20953-2	Snake Mounds	9º43.026	12º46.005	259	Box Corer	Cnidaria	Hydrozoa	Hyd
GeoB 20927-1	Buffalo Mounds	9º42.270	12º43.858	402	ROV	Porifera	Aphrocallistes sp	Aph
GeoB 20930-1	Scary Mounds	9º49.364	12º46.405	412	ROV	Porifera	Aphrocallistes sp	Aph
GeoB 20930-1	Scary Mounds	9º49.364	12º46.405	412	ROV	Porifera	Aphrocallistes sp	Aph
GeoB 20930-1	Scary Mounds	9º49.364	12º46.405	412	ROV	Porifera	Sympagella sp	Sym
GeoB 20920-1	Anna Ridge	9º44.763	12º46.929	336	ROV	Porifera	Hexactinellida	Hex
GeoB 20917-1	Valentine Mounds	9º43.769	12º42.851	473	ROV	Porifera	Demospongiae	Dem
N/A	N/A			N/A	Grab	Porifera	Porifera	Por
GeoB20920-1	Anna Ridge	9º44.763	12º46.929	336	ROV	Echinodermata	Echinus sp	Ech
GeoB20920-1	Anna Ridge	9º44.763	12º46.929	336	ROV	Echinodermata	Echinus sp	Ech
GeoB20920-1	Anna Ridge	9º44.763	12º46.929	336	ROV	Echinodermata	Echinus sp	Ech
N/A	N/A			N/A	Grab	Echinodermata	Ophiuroidea	Oph
GeoB 20904-1	Valentine Mounds	9º43.769	12º42.851	503	ROV	Echinodermata	Ophiothrix sp	Oph
GeoB 20927-1	Buffalo Mounds	9º42.270	12º43.858	402	ROV	Echinodermata	Marthasterias	Ast
GeoB 20904-1	Valentine Mounds	9º43.769	12º42.851	503	ROV	Echinodermata	Asteroidea	Ast
GeoB 20957-1	Castle Mounds	9º39.901	12º42.945	447	ROV	Echinodermata	Asteroidea	Ast
GeoB 20910-1	Twin Mounds	9º43.573	12º44.664	334	Grab	Annelida	*Eunice norvegica*	E_nor
GeoB 20904-1	Valentine Mounds	9º43.769	12º42.851	503	ROV	Annelida	*Eunice norvegica*	E_nor

GeoB 20904-1	Valentine Mounds	9º43.769	12º42.851	503	ROV	Annelida	Polynoidae	Poly
GeoB 20920-1	Anna Ridge	9º44.763	12º46.929	336	ROV	Arthropoda	Munida sp	Mun
GeoB 20913-1	Anna Ridge	9º47.296	12º46.401	307	Grab	Arthropoda	Munida sp	Mun
GeoB 20920-1	Anna Ridge	9º44.763	12º46.929	336	ROV	Chordata	Myctophidae	Mycto
GeoB 20920-1	Anna Ridge	9º44.763	12º46.929	336	ROV	Chordata	Myctophidae	Mycto
GeoB 20920-1	Anna Ridge	9º44.763	12º46.929	336	ROV	Chordata	Myctophidae	Mycto
GeoB 209332	Scary Mounds	9º49.336	12º46.565	345	Grab	Food Source	Sediment-345m	Sed345
GeoB20953-2	Snake Mounds	9º43.026	12º46.005	259	Box Corer	Food Source	Sediment-259m	Sed259
GeoB 20921-1	Anna Ridge	9º46.14	12º45.96	342	Sed trap	Food Source	Sediment trap-342m	SPOMtrap342
GeoB 20916-1	Valentine Mounds	9º43.660	12º42.09	526	Sed trap	Food Source	Sediment trap-526m	SPOMtrap526
GeoB20921-1	Anna Ridge	9º46.14	12º45.96	342	McLane pump	Food Source	McLane SPOM-342m	SPOM342
GeoB20940-1	Valentine Mounds	9º43.8	12º42.12	532	McLane pump	Food Source	McLane SPOM-532m	SPOM532

Supplementary Figure S2. Different methods used to find out optimal Number of Clusters for k-means analysis. Calculated with the "*nbclust*" package.

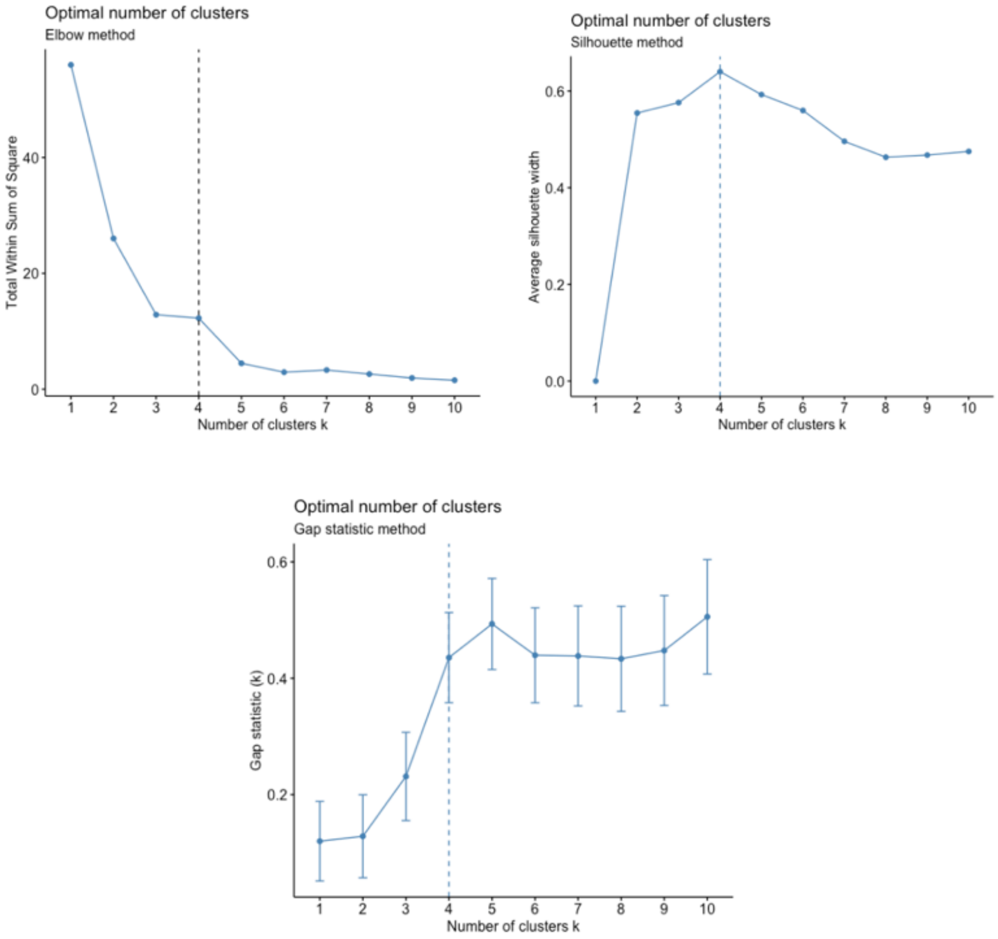

Supplement: Supplementary file 1 — Supplementary Information. [file 41598_2023_37035_MOESM1_ESM.pdf]
